# Clinical Outcomes of Drug-Coated Balloon in Coronary Patients with and without Diabetes Mellitus: A Multicenter, Propensity Score Study

**DOI:** 10.1155/2021/5495219

**Published:** 2021-07-29

**Authors:** Liang Pan, Wenjie Lu, Zhanying Han, Sancong Pan, Xi Wang, Yingguang Shan, Xule Wang, Xiaolin Zheng, Ran Li, Yanjun Zhou, Peng Qin, Qiangwei Shi, Shuai Zhou, Wencai Zhang, Sen Guo, Peisheng Zhang, Xiaofei Qin, Guoju Sun, Zhongsheng Qin, Zhenwen Huang, Chunguang Qiu

**Affiliations:** ^1^Department of Cardiovascular Medicine, The First Affiliated Hospital of Zhengzhou University, China; ^2^Department of Cardiovascular Medicine, Jincheng People's Hospital, China; ^3^Department of Geriatric Cardiology, The First Affiliated Hospital of Zhengzhou University, China; ^4^Department of Cardiology, The Fifth Affiliated Hospital of Zhengzhou University, China

## Abstract

**Background:**

Relative to nondiabetic patients, percutaneous coronary intervention (PCI) in patients with diabetes mellitus (DM) is associated with inferior clinical outcomes. We aimed to evaluate the outcomes of drug-coated balloon (DCB) in diabetic versus nondiabetic patients.

**Methods and Results:**

In this observational, prospective, multicenter study, we compared the outcomes of patients with and without DM after undergoing PCI with DCBs. Target lesion failure (TLF) was analyzed as primary endpoint. Secondary endpoints were the rates of target lesion revascularization (TLR), major adverse cardiovascular events (MACE), cardiac death, myocardial infarction (MI), and any revascularization. Propensity score matching was used to assemble a cohort of patients with similar baseline characteristics. Among 2,306 eligible patients, 578 with DM and 578 without DM had similar propensity scores and were included in the analyses. During follow-up (366 ± 46 days), compared with DM patients, patients without DM were associated with a lower yearly incidence of TLF (2.77% vs. 5.36%; OR, 1.991; 95% CI, 1.077 to 3.681; *P* = 0.025) and TLR (1.90% vs. 4.15%; OR, 2.233; 95% CI, 1.083 to 4.602; *P* = 0.026). No significant differences were observed with regards to rates of MACE (OR: 1.580, 95% CI: 0.912-2.735; *P* = 0.100), cardiac death (OR: 1.608, 95% CI: 0.523-4.946; *P* = 0.403), MI (OR: 4.042, 95% CI: 0.855-19.117; *P* = 0.057), and any revascularization (OR: 1.534, 95% CI: 0.983-2.393; *P* = 0.058).

**Conclusions:**

Diabetic patients experience higher TLF and TLR rates following DCB angioplasty without substantial increase in the risk of MACE, cardiac death, MI, or revascularization.

## 1. Introduction

Patients with diabetes mellitus (DM) are at increased risk of coronary artery disease (CAD). DM patients often present with a combination of diffuse coronary lesions and small vessel disease, indicating difficult stent delivery and high restenosis rates postpercutaneous coronary intervention (PCI). Regarding coronary revascularization, recent technological advances have expanded PCI use to more complex lesions [1, 2], especially drug eluting stents (DES), which have markedly reduced the rate of restenosis and repeat revascularization [3, 4]. However, CAD morbidity and mortality in DM patients remain high, even with current use of DES [5, 6].

Drug-coated balloon (DCB) is effective in treating instent restenosis (ISR) [7, 8]. Various clinical studies have revealed its effectiveness against de novo coronary lesions [9–11], especially in small vessel disease. Differences in study results are mainly due to differences in study approach, which may use the “DCB-only” or “hybrid” strategy. It also preserves access for future coronary artery bypass grafting (CABG).

To date, no studies have investigated the use of DCB angioplasty-only in diabetic patients with coronary artery disease. Here, we evaluated the outcomes of DCBs in diabetic versus nondiabetic patients.

## 2. Methods

### 2.1. Study Population

**Figure 1:**
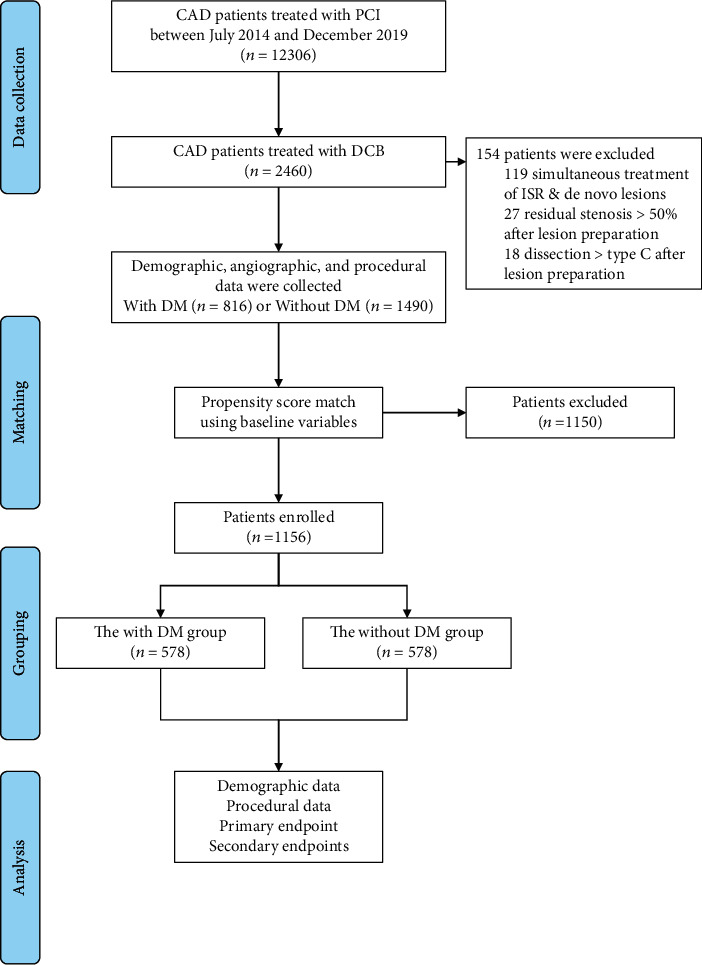
Study population. CAD: coronary artery disease; PCI: percutaneous coronary intervention; DCB: drug-coated balloon; DM: diabetes mellitus; ISR: instent restenosis.

Patients were retrospectively enrolled at 3 Chinese centers from July 2014 to December 2019. Only patients with coronary vessel lesions sized between 2.0 and 4.0 mm, whether de novo or instent restenosis, and were eligible for the study. The exclusion criteria were ≥ type C dissection or >30% residual stenosis after lesion preparation, simultaneous ISR treatment and de novo lesions, revascularization within 1 month prior to index procedure, and unstable hemodynamics or cardiogenic shock (Figure 1).

Patients were given 300 mg aspirin before intervention, unless they were on long-term aspirin treatment. Clopidogrel or ticagrelor was administered at loading doses of 600 and 180 mg, respectively. Patients who underwent DCB only were put on dual antiplatelet therapy (DAPT) for at least 1 month after the procedure. Those receiving DCB and stent implantation received DAPT in accordance with established guidelines [12, 13]. Patients with contraindications or known hypersensitivity to DAPT, heparin, paclitaxel, or limus; women with childbearing potential; and those with a life expectancy below a year were excluded. Ethical approval for the study was granted by the First Affiliated Hospital of Zhengzhou University Institutional Review Board/Ethics Committee. All patients gave written informed consent. Data were collected using a common electronic case report form.

### 2.2. Study Procedure

During intervention, special emphasis was paid to adequate lesion preparation prior to DCB treatment as recommended in the latest expert consensus [14]. Predilatation with semicompliant balloon, noncompliant balloon, scoring balloon, or cutting balloon with a balloon-to-vessel ratio of 0.8-1.0 was mandatory (0.8-1.0 for de novo lesions and 1.0 for ISR lesions). Next, DCB angioplasty was done only in the absence of a major, flow-limiting dissection (≥ type C according to the NHLBI classification [15] and where residual stenosis was ≤30% based on at least 2 perpendicular angiographic views). In this study, DCB had a paclitaxel/iopromide matrix coating (SeQuent™ Please, B. Braun, Melsungen, Germany). To avoid geographic mismatch, DCB catheter length was designed to exceed that of the target lesion by at least 2 mm. DCB diameters were adapted to reference vessel diameters with a balloon-to-vessel ratio of 0.8-1.0. Recommended inflation time was at least 30 seconds at >7 bars. New-generation DESs were implanted if DCB-only outcomes were unsatisfactory due to severe residual stenosis or dissections.

### 2.3. Clinical Endpoints and Definitions

The study's primary outcome was one-year target lesion failure (TLF), a composite of cardiac death, target vessel myocardial infarction (MI), and target lesion revascularization (TLR). Various secondary outcomes were evaluated, including the rates of TLR, major adverse cardiovascular events (MACE, defined as the composite outcome of cardiac death, myocardial infarction (MI), and target vessel revascularization (TVR)), cardiac death, MI, and repeat revascularization (including PCI and CABG). Cardiac death is defined as death resulting from cardiovascular causes. And undetermined cause of death is defined as a death not attributable to any other category because of the absence of any relevant source documents. Such deaths will be classified as cardiovascular for end point determination [16, 17]. MI was defined by typical clinical symptoms, ECG changes, and/or elevated cardiac troponin values with at least one value above the 99^th^ percentile upper reference limit (type 4b or 4c MI, except for perioperative MI) [18]. Patients' follow-up was by telephone or outpatient visits at 12 months.

### 2.4. Statistical Analysis

Data analysis was done using *R* statistics packages (http://www.r-project.org) and Empower (*R*) (http://www.empowerstats.com, X & Y Solutions Inc.). Categorical variables are presented as frequencies (percentages) and continuous variables as mean ± SD. Comparisons between patients with and without DM were done using Fisher's exact test for each variable. Mann–Whitney Wilcox nonparametric tests were used for continuous variables. Given the baseline characteristic differences between eligible participants in the 2 groups of the observational study, a 1 : 1 propensity score matching (PSM) was used to select patients with comparable baseline data. After evaluation of covariates that were clinically and/or statistically associated with the treatment group and removal of repeatedly defined or collinear variables, including baseline characteristics, risk factors, clinical conditions at admission, and treatment during operation, 12 variables (age, sex, hypertension, hyperlipidemia, renal insufficiency, acute coronary syndrome, family history of CAD, smoking history, PCI history, MI history, CABG history, and left ventricular ejection fraction) were included in the propensity score matching model using greedy nearest neighbor matching without replacement and a caliper of 0.02. Analyses of primary and secondary outcomes in the presence or absence of DM were done for the entire group and for the propensity matched cohort. Outcomes were compared using a log-rank test and presented as Kaplan-Meier curves. For all analyses reported, *P* values were 2-sided, and *P* < 0.05 indicated statistical significance.

## 3. Results

### 3.1. Patient Population

A total of 2,306 PCI patients treated with DCB met our inclusion and exclusion criteria (Figure 1). Of these 816 patients, (35.38%) had DM. Baseline characteristics are shown in Table 1. After matching, 578 patients were selected for each group. Statistical differences between the groups with regards to age, hypertension, hyperlipidemia, renal insufficiency, PCI history, and LVEF were reduced upon patient matching.

### 3.2. PCI-Related Characteristics

Procedural baseline features are shown on Table 2. There were 2,660 lesions before matching, of which 424 (15.94%) were ISR and 2,236 (84.06%) were de novo lesions. After matching, 1,318 lesions remained, 649 in non-DM patients, and 669 in DM patients. A small proportion of patients (1.85% non-DM and 1.05% DM) also underwent DCB angioplasty in the left main and bypass graft vessel. Lesion preparation (predilatation with plain balloons, scoring balloons, cutting balloons, noncompliant balloons, or rotational atherectomy) was done for all lesions. DCB use was similar in the 2 groups. 1.11 ± 0.36 DCBs were used per lesion, the mean diameter was 2.75 ± 0.47 vs. 2.75 ± 0.48 mm, the total length was 25.03 ± 12.47 vs. 25.03 ± 12.16 mm, and inflation pressure was 8.26 ± 2.93 vs. 8.23 ± 1.35 bars (non-DM vs. DM group). The bailout stenting rate was low in the non-DM (3.08%) and DM group (3.59%).

### 3.3. Clinical Outcomes

**Figure 2:**
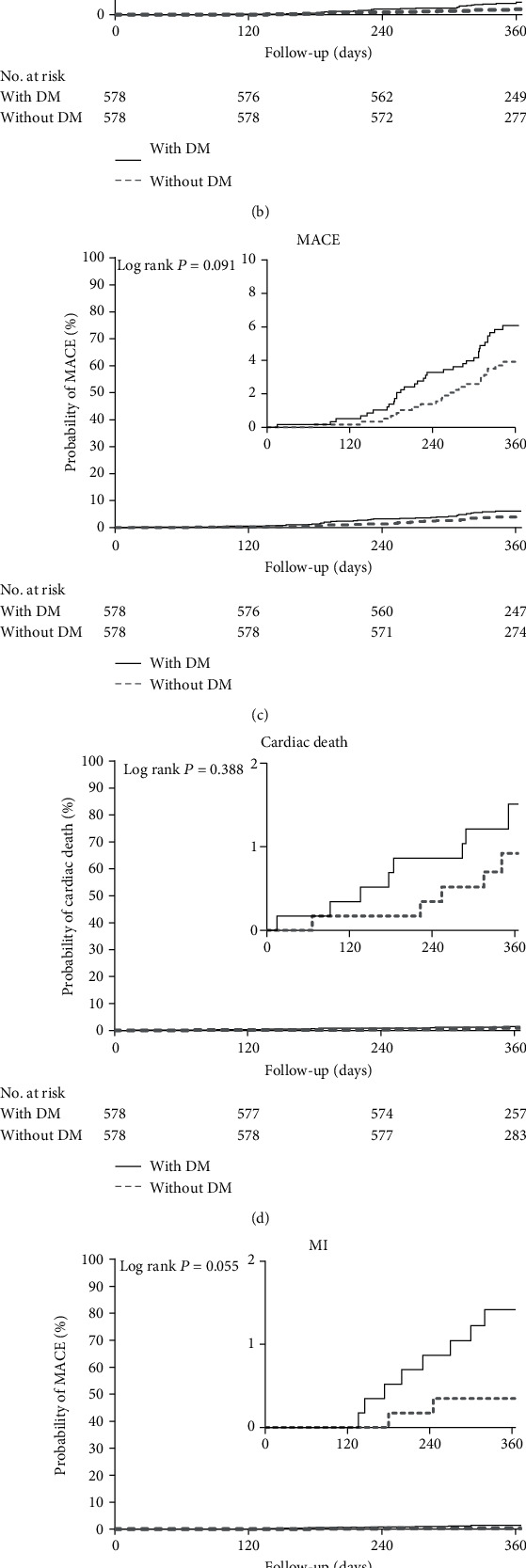
Cumulative risks of the study outcomes in the matched cohort (a), TLF (b), TLR (c), MACE (d), cardiac death (e), And MI (f). In any revascularization, DM: diabetes mellitus; TLF: target lesion failure; TLR: target lesion revascularization; MACE: major adverse cardiovascular events; MI: myocardial infraction. In each panel, the inset shows the same data on an enlarged *y* axis.

Overall population follow-up for a mean of 366 days revealed a TLF incidence rates of 4.53% and 2.42% in diabetic and nondiabetic patients, respectively (OR: 1.918, 95% CI: 1.203-3.060; *P* = 0.005). After PSM, relative to DM-patients, non-DM patients exhibited lower yearly TLF incidence (5.36% and 2.77%, respectively, OR: 1.991, 95% CI: 1.077-3.681; *P* = 0.025; log rank *P* = 0.023) (Table 3 and Figure 2(a)). Results were largely similar before and after PSM.

After matching, Kaplan-Meier analysis (Table 3, Figures 2(b)–2(f)) revealed that the cumulative rate of TLR was higher in the DM group at 1 year (with DM: 4.15% vs. without DM: 1.90%; OR, 2.233; 95% CI, 1.083 to 4.602; *P* = 0.026; log rank *P* = 0.022). MACE incidence was lower in the non-DM (3.81%) vs. DM (5.88%) group but without statistical difference (OR: 1.580; 95% CI: 0.912-2.735; *P* = 0.100; log rank *P* = 0.091). Additionally, in the non-DM vs DM group, there were no statistical differences with regards to incidence of cardiac death (OR: 1.608, 95% CI: 0.523-4.946, *P* = 0.403; log rank *P* = 0.388), MI (OR: 4.042, 95% CI: 0.855-19.117, *P* = 0.057; log rank *P* = 0.055), or any revascularization (OR: 1.534, 95% CI: 0.983-2.393, *P* = 0.058; log rank *P* = 0.052). However, the incidence of MI or any revascularization in the DM group trended upward over time.

### 3.4. Sensitivity Analysis

In the overall population, TLF exhibited good consistency before and after PSM. The incidence rates of TLR (OR: 2.251, 95% CI: 1.302-3.893), MACE (OR: 1.830, 95% CI: 1.197-2.798), MI (OR: 6.150, 95% CI: 1.688-22.409), and any revascularization (OR: 1.581, 95% CI: 1.132-2.209) in the diabetic group were higher than in the nondiabetic group (all *P* < 0.05, Table 3). Additionally, binary logistic regression analysis of TLF in the overall population revealed that DM increased TLF risk (OR: 1.721, 95% CI: 1.012-2.927, *P* = 0.045).

## 4. Discussion

In this observational study, we evaluated the outcomes of PCI with DCB in diabetic versus nondiabetic patients treated for CAD at three participating centers. Our data suggest that relative to nondiabetic patients, diabetic patients treated with PCI with DCB exhibit higher incidence of TLF and TLR than nondiabetic patients. These findings enhance our current understanding of the safety and efficacy of DCB in all comers in contemporary clinical practice. However, the incidence rates of MACE, cardiac death, and any revascularization in the 2 groups were similar, suggesting that DCB angioplasty can serve as the default treatment option for such patients. Because our results are based on matching propensity scores, our findings are unlikely to result from negative confounding. Moreover, reliability of these findings was validated via sensitivity analysis methods like subgroup analysis.

CAD in the presence of DM has unique characteristics. The higher risk of ISR in patients with DM is secondary to complex pathophysiological mechanism, including endothelial dysfunction, serious vascular inflammation, high activated platelet levels, and higher levels of advanced end products of glycosylation [19, 20]. ISR risk within first-generation DES is higher due to sustained drug release and the inflammatory effects of the polymer [21]. On how to explain the increased risk of stent undersizing or underexpansion and subsequent increase in stent-related complications, a past study found that when compared to non-DM patients, diabetic patients treated with DES have greater residual plaque burden throughout the reference segment [22].

Development of second-generation DES with biocompatible polymers and thinner struts has improved diabetic patients' outcomes upon PCI [23]. However, diabetes remains an independent predictor of major adverse events [24]. In a recent study of 1,919 patients who received PCI with 2 different new-generation DES, diabetic patients had higher target lesion failure (TLF) risk (cardiac death, target vessel myocardial infarction, or ischemia-driven TLR) relative to non-DM patients (7.8% vs. 4.2%, *P* = 0.002), mainly due to a higher TLR rate (4.5% vs. 2.0%, *P* = 0.002) [6]. In diabetic patients, DCB has various advantages over DES, including even distribution of the antiproliferative drug along the vessel wall, resulting in better positive remodeling [25, 26]. In addition, local inflammatory reactions in diabetic patients are often severe, and DCB releases higher drug concentrations in the short-term, which is more conducive to inhibit inflammatory reactions. Although the new-generation DES uses more biocompatible or absorbable polymer, the continuous stimulation of the stent struts may cause the local inflammation to persist.

To our knowledge, no studies have investigated the use of DCB-only strategy in diabetic patients with CAD. The PEPCAD IV DM study randomly allocated 84 diabetic patients with significantly stenosed native coronary arteries to a paclitaxel-coated PTCA-balloon SeQuent™ Please, followed by deployment of the cobalt-chromium stent Coroflex™ Blue treatment group or to a paclitaxel-eluting stent Taxus™ Liberté treatment group [11]. At 9 months angiographic follow-up, the primary outcome of mean insegment late lumen loss was 0.37 ± 0.59 mm in the DCB + bare metal stents (BMS) group vs. 0.35 ± 0.63 mm in the DES group (*P* = 0.89). MACE rates were also similar in both groups (13.3% in DCB vs. 15.4% in DES, *P* = 0.96). Of note, although pr-dilatation was recommended in the study protocol, it was done in only 31.1% of DCB + BMS-treated patients. Additionally, that study was terminated prematurely due to slow patient enrolment. Further data on treating DM patients with DCB came from the DiabEtic Argentina Registry (DEAR), an observational, prospective, nonrandomized, open-label study [27]. A total of 92 patients at 3 centers were enrolled for DCB angioplasty with the DIOR™ II DCB. Subsequent BMS implantation was performed in 96% of the patients. These patients were compared with diabetic patients enrolled in other clinical trials treated at the same centers with BMS (*n* = 96) or DES (*n* = 29) implantation. At 1 year, MACE rates in the DCB-treated group were significantly lower relative to the BMS-treated group and similar to those of the DES-treated group (13.2% for DCB vs. 32.3% for BMS vs. 18.6% for DES). No angiographic measures were collected. Furthermore, a recent comprehensive meta-analysis compared DCB vs. DES outcomes in PCI against de novo CAD in diabetic patients [28]. Three studies involving 378 patients (440 lesions) were included. During a 17.3 ± 11.3 months follow-up, DCB's MACE risk (OR: 0.63, 95% CI: 0.36-1.12, *P* = 0.11), TLR (OR: 0.51, 95% CI: 0.25-1.06, *P* = 0.07), binary restenosis (OR: 0.42, 95% CI: 0.09-1.92, *P* = 0.26), and LLL (mean difference, -0.13 mm, 95% CI: -0.41-0.14, *P* = 0.34) were similar to those of DES. Finally, it is inferred that in diabetic patients with de novo coronary lesions undergoing PCI, DCBs are associated with similar outcomes relative to first-generation DES, with a signal toward potential benefit in lowering target lesion revascularization. In the early stage, BMS implantation after DCB angioplasty was to avoid safety problems caused by excessive local antiproliferative drug concentration. However, the safety of DES implantation at the same location of DCB angioplasty has been verified in those patients with bailout DES deployment [29, 30]. What needs to be pointed out is that whether it is the combination of DES + DCB or DES only, the incidence rates of ISR, stent thrombosis, and other adverse events that are lower than these in combination of DCB and BMS. In addition, the control groups of these studies are all 1st-generation DES. Although antiproliferative drug of DCB and 1st-generation DES is the same, as far as current clinical practice is concerned, the significance of such a comparison is relatively small.

Here, relative to non-DM patients, PCI with DCB in diabetic patients was associated with higher TLF risk before and after PSM. And binary logistic regression analysis of TLF in the overall population revealed that DM increased TLF risk. Most importantly, the incidences of TLR, MACE, cardiac death, and any revascularization in DCB-treated diabetic patients were much lower relative to DES-treated patients reported in past studies. If the answer is yes, the choice between DCB and DES, even PCI and CABG, should be made a great change.

### 4.1. Limitation

Because this was a nonrandomized, observational study, it suffers from potential selection and ascertainment bias despite our robust propensity score matching. Additionally, as part of PCI strategy, 32.6% of the patients received DES implantation in a different coronary artery in the same operation, which may affect the occurrence of clinical events. Lastly, this study only evaluated DCB application in patients with coronary heart disease and diabetes and could not be compared to DES over a same period. Thus, a prospective randomized clinical trial comparing the use of DCB and DES in diabetic coronary heart disease patients is highly warranted as it may have important clinical significance.

## 5. Conclusion

In conclusion, our findings suggest that relative to non-DM patients, DM patients experience higher TLF and TLR rates upon DCB angioplasty. However, there was no substantial increase in the risk of MACE, cardiac death, MI, and any revascularization attributable to DM.

## Figures and Tables

**Table 1:** Demographic characteristics before and after propensity score matching.^∗^

Variable	All patients			Propensity matched sample		
	Without DM	With DM	*P* value	Without DM	With DM	*P* value
Number of patients	1490	816		578	578	
Age (years)	58.71 ± 11.17	61.26 ± 10.54	<0.001	59.99 ± 11.26	61.03 ± 10.34	0.237
Sex (male)	1077 (72.28%)	567 (69.49%)	0.156	409 (70.76%)	404 (69.90%)	0.748
Hypertension	697 (46.78%)	487 (59.68%)	<0.001	337 (58.30%)	359 (62.11%)	0.186
Hyperlipidemia	391 (26.24%)	309 (37.87%)	<0.001	200 (34.60%)	219 (37.89%)	0.245
History of smoking	490 (32.89%)	267 (32.72%)	0.936	207 (35.81%)	202 (34.95%)	0.758
Renal insufficiency	52 (3.49%)	63 (7.72%)	<0.001	28 (4.84%)	33 (5.71%)	0.511
Acute coronary syndrome	1001 (67.18%)	572 (70.10%)	0.150	435 (75.26%)	430 (74.39%)	0.735
Previous MI history	146 (9.80%)	89 (10.91%)	0.400	63 (10.90%)	65 (11.25%)	0.851
Previous PCI history	406 (27.25%)	292 (35.78%)	<0.001	190 (32.87%)	210 (36.33%)	0.216
Previous CABG history	28 (1.88%)	19 (2.33%)	0.465	12 (2.08%)	12 (2.08%)	1.000
Family history of CAD	265 (17.79%)	166 (20.34%)	0.132	127 (21.97%)	122 (21.11%)	0.721
Previous stroke history	146 (9.80%)	204 (25.00%)	<0.001	99 (17.13%)	121 (20.93%)	0.099
Peripheral artery disease	119 (7.99%)	177 (21.69%)	<0.001	81 (14.01%)	104 (17.99%)	0.065
LVEF	59.99 ± 7.55	58.95 ± 7.68	0.003	59.18 ± 7.61	59.25 ± 7.46	0.596
Other vessel treated by DES only	487 (32.68%)	279 (34.19%)	0.463	195 (33.74%)	196 (33.91%)	0.950

^∗^Plus–minus values are means ± SD. DM: diabetes mellitus; MI: myocardial infarction; PCI: percutaneous coronary intervention; CABG: coronary artery bypass grafting; CAD: coronary artery disease; LVEF: left ventricular ejection fraction; DES: drug-eluting stent.

**Table 2:** Procedural characteristics before and after propensity score matching.^∗^

Variable	All patients			Propensity matched sample		
	Without DM	With DM	*P* value	Without DM	With DM	*P* value
Number of lesions	1704	956		649	669	
Lesion type			0.610			0.587
Instent restenosis	267 (15.67%)	157 (16.42%)		116 (17.87%)	112 (16.74%)	
De novo lesions	1437 (84.33%)	799 (83.58%)		533 (82.13%)	557 (83.26%)	
Treated vessel			0.541			0.729
Left anterior descending coronary artery	775 (45.48%)	410 (42.89%)		274 (42.22%)	284 (42.45%)	
Left circumflex coronary artery	545 (31.98%)	310 (32.43%)		200 (30.82%)	215 (32.14%)	
Left main coronary artery	9 (0.53%)	3 (0.31%)		5 (0.77%)	2 (0.30%)	
Right coronary artery	363 (21.30%)	226 (23.64%)		163 (25.12%)	163 (24.36%)	
Bypass graft	12 (0.70%)	7 (0.73%)		7 (1.08%)	5 (0.75%)	
Number of lesions treated by DCB (per patient)			0.417			0.372
1	1294 (86.85%)	689 (84.44%)		511 (88.41%)	497 (85.99%)	
2	180 (12.08%)	115 (14.09%)		63 (10.90%)	72 (12.46%)	
3	14 (0.94%)	11 (1.35%)		4 (0.69%)	8 (1.38%)	
4	2 (0.13%)	1 (0.12%)		0 (0.00%)	1 (0.17%)	
Total occlusion	201 (11.80%)	116 (12.13%)	0.796	77 (11.86%)	78 (11.66%)	0.908
Intracoronary thrombus	8 (0.47%)	4 (0.42%)	0.850	4 (0.62%)	3 (0.45%)	0.722
Diffuse vessel disease	383 (22.48%)	208 (21.76%)	0.669	162 (24.96%)	151 (22.57%)	0.308
Ostial lesion	310 (18.19%)	169 (17.68%)	0.740	105 (16.18%)	118 (17.64%)	0.480
Bifurcation lesion	552 (32.39%)	292 (30.54%)	0.325	201 (30.97%)	207 (30.94%)	0.991
Lesion preparation	1704 (100%)	956 (100%)		649 (100%)	669 (100%)	
Semicompliant balloon	1119 (65.67%)	667 (69.77%)	0.031	446 (68.72%)	475 (71.00%)	0.367
NSE	459 (26.94%)	264 (27.62%)	0.706	197 (30.35%)	182 (27.20%)	0.207
Cutting balloon	521 (30.58%)	282 (29.50%)	0.561	186 (28.66%)	202 (30.19%)	0.541
DWB	67 (3.93%)	39 (4.08%)	0.852	20 (3.08%)	28 (4.19%)	0.285
Noncompliant balloon	454 (26.64%)	280 (29.29%)	0.143	168 (25.89%)	190 (28.40%)	0.305
ROTA	28 (1.64%)	26 (2.72%)	0.059	12 (1.85%)	17 (2.54%)	0.392
Number of DCBs used (per lesion)	1.10 ± 0.36	1.11 ± 0.36	0.603	1.11 ± 0.36	1.11 ± 0.36	0.716
Mean DCB diameter (mm)	2.79 ± 0.47	2.75 ± 0.47	0.026	2.75 ± 0.47	2.75 ± 0.48	0.819
Length of DCB balloon (mm)	24.50 ± 12.34	24.98 ± 12.46	0.077	25.03 ± 12.47	25.03 ± 12.16	0.569
Inflation pressure (bar)	8.36 ± 2.84	8.32 ± 2.19	0.615	8.26 ± 2.93	8.23 ± 1.35	0.404
Bailout stenting	60 (3.52%)	38 (3.97%)	0.551	20 (3.08%)	24 (3.59%)	0.609

^∗^Plus–minus values are means ± SD. DM: diabetes mellitus; DCB: drug-coated balloon; NSE: noncompliant scoring balloon; DWB: dual wire balloon; ROTA: rotational atherectomy.

**Table 3:** Risk of primary and secondary outcomes in the propensity score-matched cohort at one-year follow-up.^∗^

Endpoint	All patients				Propensity matched sample			
	Without DM	With DM	Odds ratio (95% CI)	*P* value	Without DM	With DM	Odds ratio (95% CI)	*P* value
Number of patients	1490	816			578	578		
TLF	36 (2.42%)	37 (4.53%)	1.918 (1.203, 3.060)	0.005	16 (2.77%)	31 (5.36%)	1.991 (1.077, 3.681)	0.025
TLR	24 (1.61%)	29 (3.55%)	2.251 (1.302, 3.893)	0.003	11 (1.90%)	24 (4.15%)	2.233 (1.083, 4.602)	0.026
MACE†	45 (3.02%)	44 (5.39%)	1.830 (1.197, 2.798)	0.005	22 (3.81%)	34 (5.88%)	1.580 (0.912, 2.735)	0.100
Cardiac death	11 (0.74%)	8 (0.98%)	1.331 (0.533, 3.323)	0.539	2 (0.87%)	8 (1.38%)	1.608 (0.523, 4.946)	0.403
MI	3 (0.20%)	10 (1.23%)	6.150 (1.688, 22.409)	0.002	2 (0.35%)	8 (1.38%)	4.042 (0.855, 19.117)	0.057
Any revascularization‡	81 (5.44%)	68 (8.33%)	1.581 (1.132, 2.209)	0.007	35 (6.06%)	52 (9.00%)	1.534 (0.983, 2.393)	0.058

^∗^DM: diabetes mellitus; CI: confidence interval; TLF: target lesion failure; TLR: target lesion revascularization; MACE: major adverse cardiovascular events; MI: myocardial infraction. †MACE defined as the composite outcome of cardiac death, myocardial infarction, and target vessel revascularization. ‡Any revascularization includes any percutaneous coronary intervention and coronary artery bypass grafting.

## Data Availability

The data sets generated during and/or analysed during the current study are not publicly available but are available from the corresponding author on reasonable request.
